# Porous Crosslinked Zwitterionic Microparticles Based on Glycidyl Methacrylate and N-Vinylimidazole as Possible Drug Delivery Systems

**DOI:** 10.3390/ijms232314999

**Published:** 2022-11-30

**Authors:** Marin-Aurel Trofin, Stefania Racovita, Silvia Vasiliu, Ana-Lavinia Vasiliu, Marcela Mihai

**Affiliations:** “Petru Poni” Institute of Macromolecular Chemistry, Aleea Grigore Ghica Voda 41A, 700487 Iasi, Romania

**Keywords:** suspension polymerization, sodium monochloroacetate, betaine, tetracycline, release mechanism

## Abstract

Crosslinked porous microparticles have received great attention as drug delivery systems lately due to their unique set of properties: the capability to form various polymer–drug combinations, low immunogenicity, patient compliance and ability to release drugs in a delayed or controlled manner. Moreover, polymers with betaine groups have shown some unique features such as antifouling, antimicrobial activity, biocompatibility and strong hydration properties. Herein, novel porous zwitterionic microparticles were prepared in two stages. The first step involves the synthesis of porous microparticles based on glycidyl methacrylate, N-vinylimidazole and triethyleneglycol dimethacrylate using the suspension polymerization technique, the second step being the synthesis of zwitterionic porous microparticles by polymer–analogous reaction in presence of sodium monochloroacetate as betainization agent. Both types of microparticles were characterized structurally and morphologically by FT-IR spectroscopy, energy dispersive X-ray analysis, scanning electron microscopy, dynamic vapors sorption and mercury porosimetry. The tetracycline loading into crosslinked and zwitterionic microparticles was also performed, the maximum tetracycline loading capacities being 87 mg/g and 135 mg/g, respectively. The drug release mechanism, elucidated by various mathematical models, is controlled by both diffusion and swelling processes as a function of the zwitterionic and/or porous microparticle structure. Both types of microparticles presented antibacterial activity against the two reference strains used in this study: *Escherichia coli* and *Staphylococcus aureus*.

## 1. Introduction

Porous materials as spherical particles have received a lot of attention lately for the numerous directions in which they can be used, such as in catalytic reactions, separation processes, consumer products (such as water softeners), biomedical devices, coating additives and controlled drug release systems [1,2,3,4,5]. As a polymer matrix, both natural [6,7,8,9,10] and synthetic polymers [11,12,13,14,15] can be used for the synthesis of porous microparticles. Among the wide variety of porous microparticles, those with zwitterionic structure allow enhancement of adsorption processes to achieve improved performance in drug delivery applications due to their good biocompatibility, non-toxicity, low immunogenicity, antimicrobial activity and antifouling activity [16,17,18].

Living organisms have molecules with a zwitterionic structure (amino acids, phospholipids, DNA) that play an important role in building of different types of molecules. Starting from this finding, numerous studies on the preparation, characterization and applications of zwitterionic materials in the form of nanofibers, porous electrodes, membranes, hydrogels, monoliths for chromatography and films, as well as on the functionalization of porous substrate (silica or other polymeric materials) by coating, grafting or covalent bonding of amino acids or betaines can be found in the literature [19,20,21,22,23]. However, few studies are directed toward preparation of porous zwitterionic microparticles. For instance, Kibar and Tuncel [24] synthesized and characterized monodisperse-porous zwitterionic microbeads based on zwitterionic functional monomer, sulfopropyl-2-vinylpyridinium hydroxide and two crosslinking agents (glycerol dimethacrylate and ethylene dimethacrylate) using a modified staged shape template polymerization. These compounds can be used as stationary phases in chromatographic columns. Well-defined core–shell polymer particles with zwitterionic structure were prepared by combined distillation precipitation polymerization and click chemistry for the high-efficiency enrichment of glycopeptide [25]. Zwitterionic ion exchangers with high adsorption capacities for inorganic and organic compounds based on 4 vinylpyridine and divinylbenzene were prepared by polymer–analogous reactions [26]. Resins bearing zwitterionic groups, based on ethyl acrylate and acrylonitrile with divinylbenzene crosslinker (3% and 8%) were obtained by aminolysis with ethylenediamine or triethylenetetramine followed by a carboxymethylation reaction [27]. The sorption capacity of the resins was tested for dyes or drugs [27], as well as for heavy metal ions (Cu(II), Fe(II), Mn(II), Zn(II)) in competitive and noncompetitive conditions, from synthetic polluted water and from Tarnita-area polluted water [28], with very good sorption properties. Moreover, these zwitterionic resins were used as scaffolds for the in situ chemical reduction of loaded silver ions to obtain silver nanoparticles. The silver-loaded composite resins presented antimicrobial activity against *E. coli*, *S. aureus* and *C. albicans* strains and could be applied in biomedical area and in water cleaning [29]. In the biomedical field, the use of microparticulate systems confers some facilities: the possibility to choose the components of the microparticle structure in order to achieve a controlled release of drug, an adequate degradation of microparticles and an improvement of pharmacokinetic and pharmacodynamics properties of drugs; the properties of microparticles such as particle size, surface and porous characteristics can be modified to obtain the desired release effect; protection of active biological principle against degradation in the body, especially against enzymatic degradation; various routes of administration [30,31].

Considering the properties of porous and zwitterionic materials, the aim of this study was to develop new porous zwitterionic microparticles containing carboxybetaine moieties, which contributes to the worldwide research effort to find solutions for obtaining drug delivery systems with improved performance in terms of safety, efficiency and patient compliance that also possess antimicrobial activity. Introduction of betaine units into the structure of crosslinked microparticles can lead to the preparation of polymeric materials, with some interesting properties for their use in biomedical applications, such as (a) increased hydrophilicity, which determines the loading of higher amount of drugs; (b) can act as controlled/sustained delivery system for both hydrophobic and hydrophilic drugs; (c) sensitivity to pH change; (d) intrinsic antimicrobial properties; (e) antifouling properties [32]. Due to the presence of betaine units in the structure of crosslinked microparticles, polymeric materials can act as either a controlled or sustained delivery system for both hydrophobic and hydrophilic drugs. Moreover, the use of simple and cheap methods for the preparation of porous zwitterionic microparticles, as well as the small volume of solvents, represents an economically friendly solution with minimal pollution effect. In this respect, the current study follows some successive steps: (1) the synthesis and characterization of porous crosslinked microparticles based on glycidyl methacrylate (GMA), N-vinylimidazole (NVI) and triethyleneglycol dimethacrylate (TEGDMA); (2) the functionalization of the obtained porous microparticles in order to prepare microparticles with betaine moieties using sodium monochloroacetate; (3) the loading and release of tetracycline hydrochloride (TCH) using both types of microparticles, along with elucidation of the drug release mechanism from microparticles using Higuchi, Korsmeyer–Peppas and Baker–Lonsdale mathematical models; (4) antibacterial activity evaluation of the microparticles.

## 2. Results and Discussion

The porous crosslinked zwitterionic microparticles were prepared by an efficient and facile method that combines the following steps: (1) synthesis of crosslinked porous microparticles via suspension polymerization and (2) polymer–analogous reactions carried out on crosslinked porous microparticles with an appropriate betainization agent. The choosing of the reaction system and synthesis technique was dictated by the desired outcome, namely the preparation of porous microparticles with a spherical shape, carrying the betaine units, as follows:

- GMA, a monomer with low toxicity and cheaper cost than other acrylic monomers, having two important functional groups in its chemical structure (methacrylic and epoxy groups), which may participate in polymerization or in ring opening reactions with the introduction of new functional groups [33,34];
- NVI, a monomer that may present biocompatibility, biodegradability and antibacterial activity, having an imidazole ring with a sterically unrestricted tertiary nitrogen atom, which in the presence of suitable betainization agents, via polymer–analogous reactions, lead to polymers containing betaine units [35,36];
- TEGDMA, a dimethacrylic monomer that can participate in crosslinking radical polymerization reactions due to the presence of the two double bonds in its chemical structure.

**Scheme 1:**
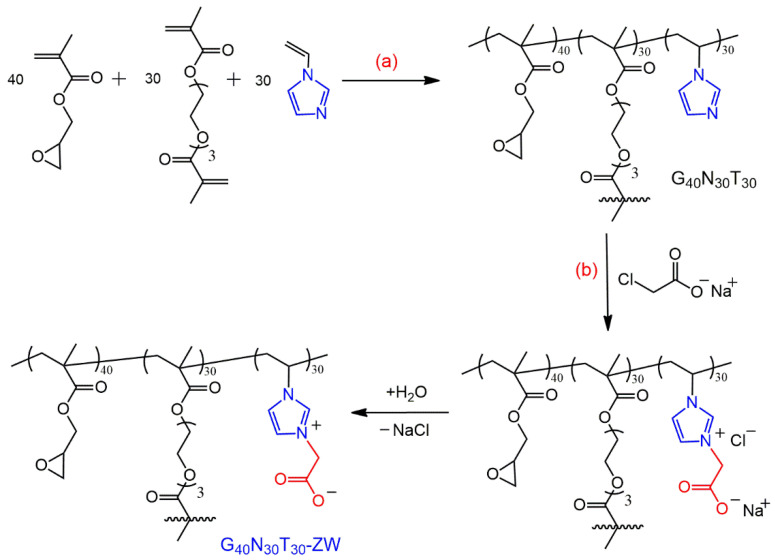
Synthesis reaction of porous crosslinked polymeric networks based on: (a) GMA, NVI and TEGDMA (G_40_N_30_T_30_) and (b) its zwitterionic derivative (G_40_N_30_T_30_-ZW).

The crosslinking radical copolymerization reaction was used to synthesize the porous microparticles based on GMA, NVI and TEGDMA (as described in the Section 3.2.1), according to the reaction shown in Scheme 1a. The synthesis trials (not described in this paper) have proven that the best microparticles, in terms of porosity, size and size distribution, were obtained with 40 moles GMA, 30 moles NVI and 30 moles TEGDMA (sample denoted as G_40_N_30_T_30_). Then, the zwitterionic microparticles (G_40_N_30_T_30_-ZW) were obtained by polymer–analogous reactions, following a nucleophilic substitution mechanism, conducted on G_40_N_30_T_30_ sample with sodium monochloroacetate (Scheme 1b). FT-IR spectroscopy was used to characterize the structures of porous and zwitterionic crosslinked microparticles, while scanning electron microscopy (SEM), mercury porosimetry and dynamic water vapor sorption (DVS) were used to characterize their morphologies.

**Figure 1:**
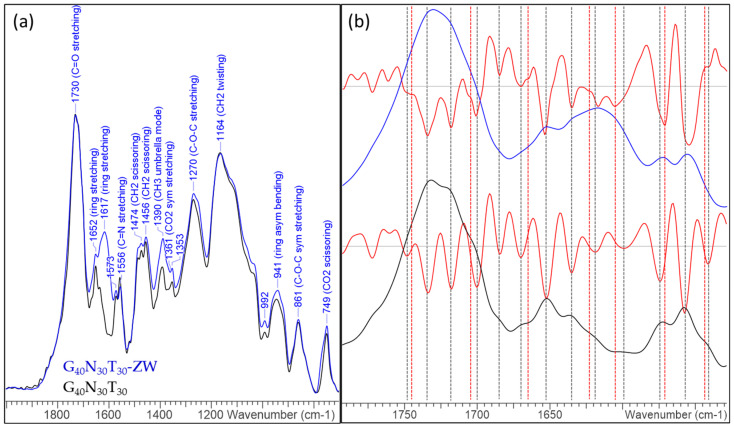
(**a**) FT-IR spectra of G_40_N_30_T_30_ and G_40_N_30_T_30_-ZW microparticles and (**b**) selected 1800–1500 cm^−1^ wavenumbers domain along with second derivative of each spectrum (doted gray lines mark the peak corresponding to G_40_N_30_T_30_ and doted red lines mark the peaks corresponding just to G_40_N_30_T_30_-ZW).

FT-IR spectroscopy offers qualitative and quantitative information about the structure of microparticles by identifying the functional groups present in their structure. The spectra of investigated microparticles (Figure 1a) have the specific bands for GMA, NVI, TEGDMA for both type of particles as automatically assigned by ACD/Spectrus Processor (Advanced Chemistry Development Inc., Toronto, Canada) as follows: C=O and COO (1730, 1361, 861 and 749 cm^−1^), CH_2_ (2968, 1474, and 1164 cm^−1^), CH_3_ (2951, 1456, and 1390 cm^−1^) and imidazole ring (1652, 1637, 1557 and 1516 cm^−1^). The large absorption band at 3700–3200 cm^−1^, found in both spectra, most probably corresponds to stretching vibrations of –OH groups, which may suggest that some epoxy groups are opened during the synthesis reaction that takes place in aqueous medium at high temperature (80–90 °C). Figure 1a also shows that the differences in the spectra appear mainly in the wavenumber domain of 1800–1500 cm^−1^; therefore this domain was zoomed and the second derivative was shown, for both G_40_N_30_T_30_ and G_40_N_30_T_30_-ZW microparticles, in Figure 1b. Thus, the second derivative curves emphasize the peaks’ contribution to the larger peaks, as marked in Figure 1b with dotted grey lines. After betainization reaction, some peaks shifted, marked with dotted red lines, and are characteristic of C-O and C=O stretching (1745 and 1706 cm^−1^) and for C=N bonds and stretching of imidazole ring (1665, 1624 and 1571 cm^−1^), thus proving the betainization reaction’s success.

**Figure 2:**
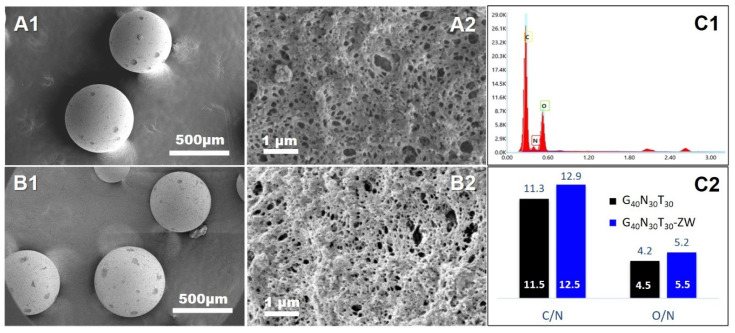
Overall and surface SEM images of G_40_N_30_T_30_ (**A1**,**A2**) and G_40_N_30_T_30_-ZW (**B1**,**B2**) microparticles; EDAX analysis as spectrum survey of G_40_N_30_T_30_ (**C1**) and both samples’ elements atomic ratio as compared to the calculated ones (white digits) (**C2**).

The morphology of the porous particles and them changes that have occurred as a result of betainization have been revealed using SEM, the images providing information about the shape, size, and surface morphology of microparticles, whereas energy dispersive X-ray analysis (EDAX) gives qualitative and quantitative information on the atomic content on the investigated samples (Figure 2).

SEM images reveal that the porous particles are spherically shaped and the betainization reaction was achieved without changing their shape (Figure 2(A1,B1)). Nevertheless, some small modification to the microparticles’ pore surface morphology can be noticed (Figure 2(A2,B2)), the presence of pores being clearly evidenced in both types of microparticles. Elemental semi-quantitative analysis, followed by EDAX, evidenced the presence of carbon, oxygen and nitrogen on the chemical structure of the porous crosslinked microparticles (Figure 2(C1)) and on the zwitterionic particles (not shown here). The increases in the values of the C/N (from 11.3 to 12.9) and O/N (from 4.2 to 5.2) atomic ratios (Figure 2(C2)) in G_40_N_30_T_30_-ZW as compared to G_40_N_30_T_30_ were assigned to the presence of the newly introduced carboxylate groups, further confirming that the betainization reaction took place. Furthermore, the obtained experimental values for these atomic ratios are close to the theoretical ones (C/N = 11.5 and 12.5, and O/N = 4.5 and 5.5, for G_40_N_30_T_30_ and G_40_N_30_T_30_-ZW, respectively) additionally confirming that the reactions took place almost quantitatively.

**Figure 3:**
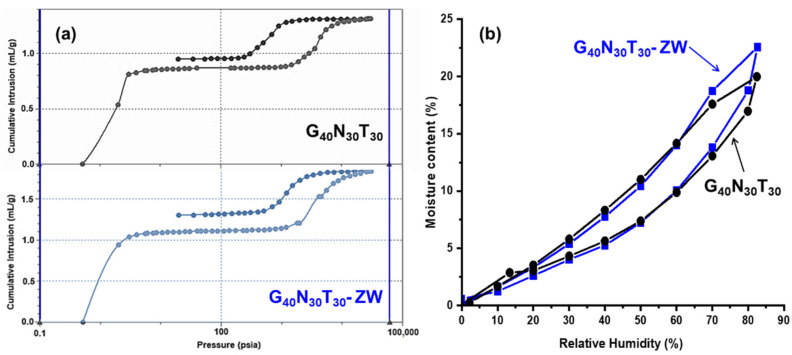
Sorption–desorption isotherms for G_40_N_30_T_30_ and G_40_N_30_T_30_-ZW microparticles determined by (**a**) mercury porosimetry; (**b**) dynamic water vapor sorption.

The most important parameters that characterize the porous structure, such as specific surface area, pore volume, porosity and mean pore diameter, as well as the samples swelling degree are presented in Table 1. The specific surface area was determined using two different methods (mercury porosimetry (Hg) and DVS), with the sorption–desorption isotherms for G_40_N_30_T_30_ and G_40_N_30_T_30_-ZW microparticles being presented in Figure 3.

As shown in Table 1, a betainization reaction with sodium monochloroacetate leads to an increase in pore volume and porosity as well as to a decrease in average pore diameters. These results could be explained by the penetration of the betainizing agent through the pores of the three-dimensional network and its binding to the imidazolic nitrogen atom, leading to a decrease in pore size. The values of specific surface area varied as a function of the applied measuring method, with differences being ascribed to each method’s working principle. Thus, DVS is a gravimetric technique that measures the water uptake as a function of relative humidity of the environment at equilibrium conditions [37], while mercury porosimetry is an analytical technique based on the pressure-dependent intrusion of mercury, as a non-wetting liquid, into a porous material. The shape of sorption isotherms provides information on the sorption mechanism of a liquid onto solids; according to IUPAC classification, physisorption isotherms are classified into six types [38].

Taking into account the shapes of the water sorption and mercury intrusion curves presented in Figure 3, these can be associated with isotherms of type IVa, which is characteristic of porous materials. The H_2_(b) hysteresis loop appears due to capillary condensation showing pore-blocking effects during the desorption step. This type of hysteresis occurs when the pore cavity is narrowed in comparison to their neck size distribution [39]. The larger values obtained by DVS are also related to the particles swelling capacity, which allows large amounts of water to be uptaken, values being larger for zwitterionic microparticles due to the presence of carboxylate as strongly hydrophilic groups in their structure. Also, the values of specific surface area are higher for G_40_N_30_T_30_-ZW microparticles than that of G_40_N_30_T_30_ microparticles, irrespective of the determination method emphasizing the small increase in porosity after the betainization process.

### 2.1. Antibacterial Activity

**Figure 4:**
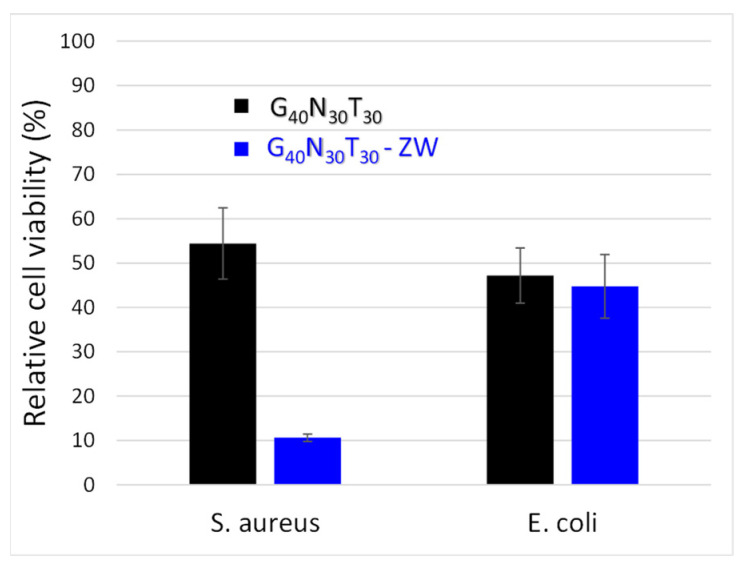
Antimicrobial activity of the tested samples against the reference strains.

The antibacterial activity of G_40_N_30_T_30_ and G_40_N_30_T_30_-ZW microparticles was studied against two reference strains, *Escherichia coli* and *Staphylococcus aureus*, the results being shown in Figure 4.

As Figure 4 shows, the tested samples presented antimicrobial activity against the two tested bacterial strains. In this particular case, both types of microparticles significantly reduced the bacterial cells’ viability, the zwitterionic porous microparticles being more efficient, especially against *S. aureus*, by decreasing the cells’ viability up to 10% when compared with the control sample. In the case of *E. coli*, no significant difference was noticed between the activities of the two types of samples, both reducing in half the cells’ viability after 24 h of incubation. In the case of *S. aureus*, it can be noticed that the zwitterionic porous microparticles were up to 90% more efficient compared with the control sample when decreasing the cells’ viability.

### 2.2. Tetracycline Loading

**Figure 5:**
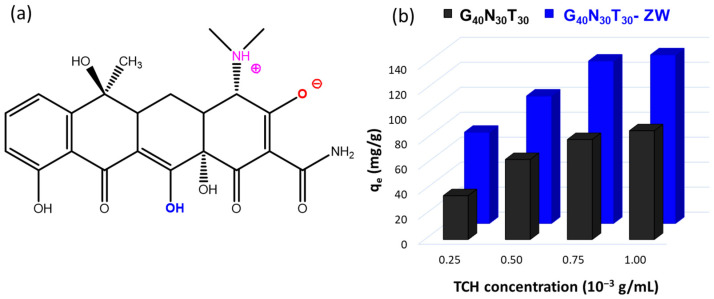
(**a**) Tetracycline (TCH) chemical structure at pH 5.5; (**b**) maximum amount of TCH sorbed onto G_40_N_30_T_30_ (black bars) and G_40_N_30_T_30_-ZW microparticles (red bars) as a function of initial TCH solution concentration.

Tetracycline is an antibiotic that belongs to a class of cyclins with bacteriostatic activity against Gram-positive and Gram-negative bacteria. Depending on the pH value, tetracycline is found in three ionized forms, namely: cationic form at pH = 3.3, anionic form at pH = 7.7 and zwitterionic form in the pH range between 3.3 and 7.7 (Figure 5a). Based on this behavior, the pH value of 5.5 was chosen for the adsorption studies of TCH onto porous crosslinked and zwitterionic microparticles. The maximum amounts of antibiotic adsorbed onto G_40_N_30_T_30_ and G_40_N_30_T_30_-ZW microparticles, as a function of initial TCH solution concentration, are shown in Figure 5b.

Figure 5b shows that the sorption capacity increases with the increase in drug concentration of the initial solution, reaching a maximum of 87 mg/g G_40_N_30_T_30_ and 135 mg/g G_40_N_30_T_30_-ZW, respectively. Moreover, irrespective of initial TCH concentration, the G_40_N_30_T_30_-ZW microparticles sorbed a higher amount of the drug compared to G_40_N_30_T_30_ microparticles, most probably due to the higher degree of swelling (Table 1), provided by (1) the presence of carboxybetaine units in their structure, facilitating an easier penetration of TCH into the pores of the microparticles, and (2) due to the electrostatic interactions between the complementary charged species.

**Figure 6:**
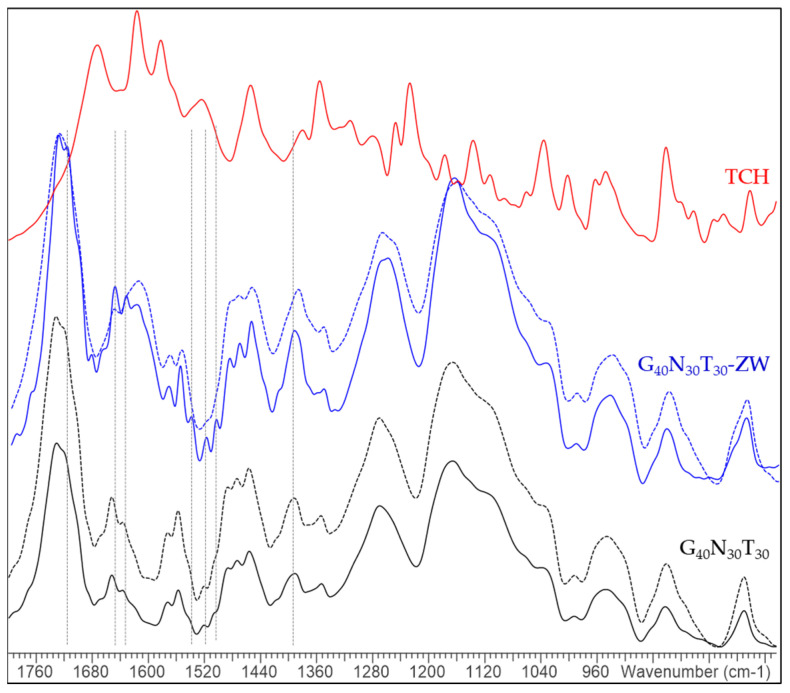
FT-IR spectra for TCH, G_40_N_30_T_30_ and G_40_N_30_T_30_-ZW microparticles loaded with TCH (straight lines) compared to initial microparticles (dotted lines).

The presence of TCH in the structure of microparticles was observed using FT-IR spectroscopy (Figure 6). FT-IR spectrum for TCH shows the following characteristic absorption bands in the selected wavenumber region (1800–700 cm^−1^): 1648–1582 cm^−1^ attributed to the stretching vibrations of –CH_3_ groups and >C=C < bonds; 1438–1357 cm^−1^ assigned to the bending vibrations of the CH bond of aromatic ring and the –CH_3_ group; the bands at 1247–1000 cm^−1^ due to in-plane and out-of-plane bending vibrations of the aromatic ring; 965 cm^−1^ attributed to the stretching vibrations of the C-N bond [40].

By comparison with the initial of G_40_N_30_T_30_-ZW microparticles (dotted lines in Figure 6), the appearance of new absorption bands in both spectra after TCH sorption, at 1670, 1635, 1541, 1520 and 1507 cm^−1^ ascribed in Figure 1a mainly to imidazole zing stretching, can be attributed to the modification of the close environment, which can be related either to TCH presence or to possible electrostatic interactions between the carboxybetaine units and complementary charged species in TCH. Moreover, the shift of absorption bands from 1391 cm^−1^ (G_40_N_30_T_30_ microparticles) and 1389 cm^−1^ (G_40_N_30_T_30_-ZW microparticles) to 1392 and 1394 cm^−1^, respectively, can be attributed to the ionic interactions between -NH_3_^+^ groups belonging to TCH and COO^−^ groups found in the structure of microparticles. On the contrary, very small peak shifting in the FT-IR spectra of G_40_N_30_T_30_ microparticles after TCH sorption were found. These findings confirm drug retention within the microparticles, suggesting a larger drug amount in the zwitterionic structures.

**Figure 7:**
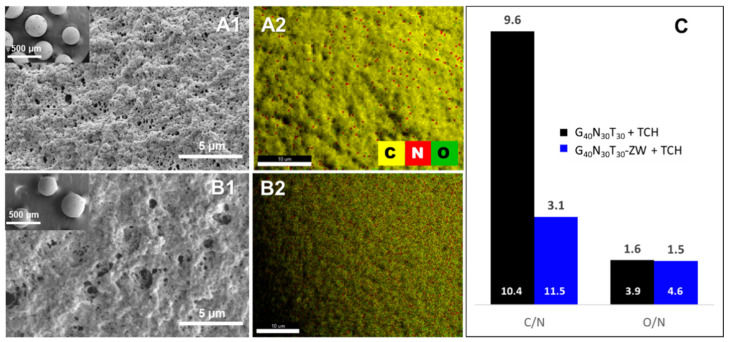
SEM images of the surface ((**A1**,**B1**); insets overall images) and EDAX element mapping (**A2**,**B2**) of G_40_N_30_T_30_ and G_40_N_30_T_30_-ZW microparticles loaded with TCH, respectively; experimental samples’ elements atomic ratio as compared to the calculated ones (white digits) (**C**); initial concentration of TCH = 1 × 10^−3^ g/mL.

SEM micrographs and EDAX element mapping performed on the microparticle surfaces provided further arguments for the sorption of TCH on the two types of macromolecular supports (Figure 7).

SEM images revealed that the porous crosslinked and zwitterionic microparticles retained their spherical shapes after the adsorption process (insets in Figure 7(A1,B1)) with smaller pores on the particles’ surface, irrespective of particle chemical structure (Figure 7(A1,B1)) as compared to initial G_40_N_30_T_30_ and G_40_N_30_T_30_-ZW microparticles (Figure 2(A2,B2)). Moreover, element-mapping of the particles’ surfaces reveals that the drug is uniformly adsorbed on the surface of microparticles, without any agglomeration or other defects being observed (Figure 7(A2,B2)). Moreover, EDAX analysis confirms the presence of TCH on the surface of porous crosslinked and zwitterionic microparticles, showing a decrease in the C/N and O/N atomic ratios after drug sorption (Figure 7C) as compared to the corresponding values obtained for microparticles before TCH sorption (Figure 2(C2)): C/N decreased from 11.3 and 12.9 to 9.6 and 3.1, and O/N from 4.2 and 5.2 to 1.6 and 1.5, for G_40_N_30_T_30_ G_40_ and G_40_N_30_T_30_-ZW microparticles, respectively. Moreover, the obtained values after TCH sorption differs significantly to that calculated taking into account the maximum TCH amount sorbed (Figure 5b), mainly when zwitterionic microparticles were used as support microparticles. Nevertheless, taking into account the higher amount sorbed in the G_40_N_30_T_30_-ZW microparticles and the SEM images in Figure 2 and Figure 6, we may assume that a large amount of TCH is included inside the microparticles by pore diffusion and kept by electrostatic interactions than on the microparticles’ surface. Moreover, the large differences between the experimental C/N values and those calculated for G_40_N_30_T_30_-ZW microparticles further demonstrate that the ionized part of TCH (Figure 4) is involved in the interaction with the carboxybetaine groups, and therefore the N element quantification on the microparticles’ surface is hindered.

### 2.3. Tetracycline Release

**Figure 8:**
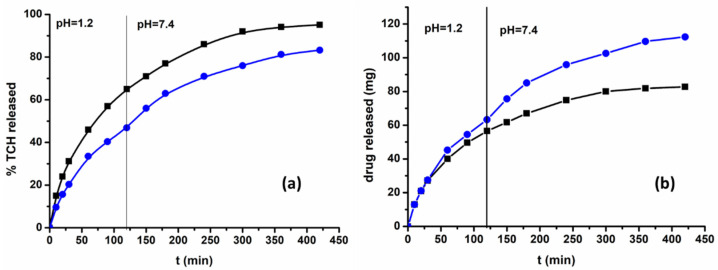
Release profiles of TCH from G_40_N_30_T_30_ (black color) and G_40_N_30_T_30_-ZW (blue color) microparticles loaded with TCH represented as (**a**) the percentage of drug released as compared to the sorbed amount and (**b**) the cumulative amount of TCH released.

The drug release process of microparticles can be influenced by several parameters: the degree of cross-linking, the morphology and density of the particulate system, the physicochemical properties of the drugs, as well as the presence of adjuvants. In vitro release also depends on pH, polarity and the presence of enzymes in the reaction medium [41]. The release of TCH from loaded G_40_N_30_T_30_ and G_40_N_30_T_30_-ZW microparticles (obtained when the initial TCH solution concentration was 1 × 10^−3^ g/mL) was studied by simulating physiologic conditions, first at pH = 1.2 for 2h and then at pH = 7.4, the release profiles being illustrated in Figure 8.

Figure 8a shows that TCH is released from G_40_N_30_T_30_ microparticles at a higher rate, the drug only being physically retained in the pores of the polymer network without the establishment of strong interactions between the macromolecular support and the biologically active principle. At the same time, the drug release of TCH-loaded G_40_N_30_T_30_-ZW microparticles occurs at a lower rate, as compared to that of G_40_N_30_T_30_ microparticles, the drug being herein retained in the pores of the three-dimensional network, not only through physical interactions but also through ionic interactions between the ionic groups belonging to TCH and the carboxybetaine ones located on the macromolecular chains of the microparticles. Nevertheless, larger drug amounts were released from TCH-loaded G_40_N_30_T_30_-ZW microparticles (Figure 8b), especially at pH of 7.4 when the electrostatic interactions are weakened by the deprotonation of amino groups in both TCH and microparticles.

The kinetics interpretation of drug release from TCH-loaded G_40_N_30_T_30_ and G_40_N_30_T_30_-ZW microparticles was performed using three mathematical models: Higuchi, Korsmeyer–Peppas and Baker–Lonsdale models.

The Higuchi model, the first mathematical model used to describe drug release from matrix systems [42], initially applied to planar systems and later extended to systems with different geometries and to porous systems [43], can be described by Equation (1):

(1) $$Q_{t}=k_{H}\cdot t^{1/2},$$

where: Q_t_ is the drug amount released at time t; k_H_ is the Higuchi dissociation constant.

The Korsmeyer–Peppas model can be used for drug release from polymeric systems [44] and is expressed by Equation (2):

(2) $$\frac{M_{t}}{M_{\infty }}=k_{r}\cdot t^{n},$$

where: M_t_/M_∞_ is the fraction of drug released at time t; k_r_ is release rate constant that is characteristic of drug–polymer interactions; n is the diffusion exponent corresponding to the release mechanism. In the case of microparticles, the values for the n parameter are:

- If n < 0.43, it corresponds to a release controlled by a diffusion process, indicating that the diffusion rate is much lower than that corresponding to the relaxation of the polymer chains. The mechanism of drug release is known as the Fick diffusion mechanism;
- If 0.43 < n < 0.85, it corresponds to an anomalous or non-Fickian diffusion mechanism where relaxation and diffusion rates have comparable values;
- If n > 0.85, the release mechanism is controlled by the relaxation phenomenon of the polymer chains and is known as super case II transport mechanism [45].

The Baker-Lonsdale model describes the release of drugs from microparticles [46], being expressed by Equation (3):

(3) $$\frac{3}{2}\left[1-{\left(1-F\right)}^{2/3}\right]-F=k_{\mathrm{BL}}\cdot t,$$

where F = M_t_/M_∞_; k_BL_ is the release constant.

**Figure 9:**
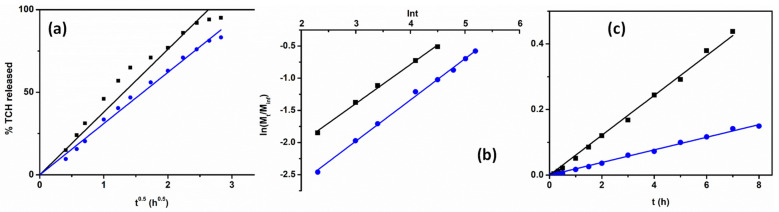
Graphical representations of (**a**) Higuchi, (**b**) Korsmeyer–Peppas and (**c**) Baker–Lonsdale mathematical models applied for drug release from G_40_N_30_T_30_ (black lines) and G_40_N_30_T_30_-ZW (blue lines) microparticles loaded with TCH.

Figure 9 shows graphical representations of the three mathematical models, the values of the kinetic parameters being included in Table 2.

As shown by the linear fits in Figure 9 and the correlation coefficients included in Table 2, all three mathematical models described well the drug release from the TCH loaded microparticles. Depending on the type of used polymer, drug release kinetics from drug delivery systems can be diffusion controlled (non-biodegradable polymers), chemically controlled (biodegradable polymers) or controlled by external factors (smart polymers that are sensitive to pH, temperature, light) [47]. The applied mathematical models revealed that the TCH release mechanism is diffusion-controlled, irrespective of the microparticles’ chemical structure. According to Table 2, the best fit models are the Korsmeyer–Peppas and Baker–Lonsdale models. Because the Higuchi model presents correlation coefficients of 0.982 and 0.986, the Baker–Lonsdale model was chosen to elucidate the tetracycline release mechanism from spherical matrices. This model is derived from the Higuchi model, and if the graphical representation is a straight line, then the drug release mechanism is controlled by the diffusion phenomenon. From Figure 9c, it can be seen that for both types of microparticle, the graphical representations of the Baker–Lonsdale model are straight lines with good correlation coefficients, which indicates that the release of tetracycline from the porous crosslinked and zwitterionic microparticles is controlled by the diffusion phenomenon. Moreover, the diffusion exponents, n, from the Korsmeyer–Peppas equation have values situated between 0.61 and 0.64 (Table 2), indicating that the TCH release mechanism is controlled both by the diffusion and by the swelling processes, being characteristic of anomalous or non-Fickian diffusion. Moreover, since the values of n are lower than 0.85, this indicates that the microparticles swelled in the release medium but do not show erosion or disintegration phenomena.

## 3. Materials and Methods

### 3.1. Materials

The reagents used in the preparation and characterization of the crosslinked microparticles were supplied by Sigma-Aldrich (Darmstadt, Germany). GMA and NVI were purified by vacuum distillation before to use. TEGDMA (crosslinked monomer), BOP (initiator), toluene (porogenic agent), PVA (M_w_ = 52,650 g/mol, degree of hydrolysis, 88), gelatin, MgCl_2_, methanol, and HCl were used as received. Sodium monochloracetate (98%), used to obtain the porous zwitterionic microparticles, was purchased from Sigma-Aldrich and used as received. TCH (M_w_ = 480.90 g/mol) was supplied by Sigma-Aldrich. Buffer solutions (pH =1.2 and 7.4) were prepared in a laboratory following standardized protocols and were used for drug release.

### 3.2. Synthesis Methods

#### 3.2.1. Synthesis of Porous Crosslinked Microparticles

Porous crosslinked microparticles were obtained by suspension polymerization technique. In the suspension polymerization method, the reaction mixture consists of two distinct phases: (a) organic phase containing monofunctional monomers (40 moles GMA and 30 moles NVI), difunctional monomer (30 moles TEGDMA), benzoyl peroxide (2.5 g BPO/100 g monomers) and porogenic agent (toluene, dilution = 0.5); (b) aqueous phase consisting of double-distilled water in which the stabilizing agent (2g PVA and gelatin/100 mL aqueous phase and 3g magnesium chloride/100 mL aqueous phase) is dissolved. The ratio between organic phase and aqueous phase was 1:9 (*v/v*). The reaction was carried out in a 250 mL cylindrical reactor equipped with Heidolph RZR 2021 mechanical stirrer (Heidolph Instruments, Schwabach, Germany), thermometer and reflux condenser. The copolymerization reactions were conducted under a nitrogen atmosphere for 8 h at 78 °C and 1 h at 90 °C, at 300 rpm stirring speed. After the polymerization process was complete, the obtained microparticles were separated by decantation, washed with warm water, dried at room temperature and then extracted with methanol in a Soxhlet apparatus to remove traces of residual monomers and porogenic agent.

#### 3.2.2. Synthesis of Porous Zwitterionic Microparticles

Porous zwitterionic microparticles were obtained through betainization reaction at tertiary nitrogen from the structure of porous crosslinked microparticles with sodium monochloroacetate. Thus, 3 g porous crosslinked microparticles were swollen in 25 mL water for 24 h at room temperature. The microparticles were separated from water by filtration and then were placed in the aqueous solution of the betainization agent (10%, *w/v*). The reaction took place under gentle stirring at 60 °C, for 120 h. Then, the obtained porous zwitterionic microparticles were filtrated and washed with bidistilled water until the absence of chlorine in the effluent, indicated by the removal of the NaCl.

### 3.3. Characterization Methods

#### 3.3.1. FT-IR Spectroscopy

FT-IR spectra of the porous crosslinked and zwitterionic microparticles were recorded on a Bruker Vertex 70 FT-IR spectrometer (Bruker, Ettlingen, Germany) in the range 4000–400 cm^−1^, collecting 124 scans, with a resolution of 2 cm^−1^, using the KBr pellet technique. To obtain the FT-IR spectra, 0.03 g of porous crosslinked and zwitterionic microparticles was mixed and ground with potassium bromide.

#### 3.3.2. SEM and EDAX

Surface morphology and elemental composition of the porous crosslinked and zwitterionic microparticles were evaluated with a Verios G4 UC scanning electron microscope (Thermo Scientific, Brno, Czech Republic). The microparticles were coated with 6 nm platinum using a Leica EM ACE 200 sputter coater (Leica microsystems, Wetzlar, Germany) to provide electrical conductivity and to prevent charge buildup during exposure to the electron beam. SEM investigations were performed in high vacuum mode using a concentric backscatter detector (CBS), working at an accelerating voltage of 5 kV. EDAX elemental mapping was conducted with an Octane Elect Super detector (Ametek, Berwyn, PA, USA).

#### 3.3.3. DVS Measurements

The behavior of the porous crosslinked and zwitterionic microparticles in the presence of moisture was studied by determining their water vapor sorption capacity in dynamic regime using a fully automated gravimetric IGAsorp device (Hidden Analytical, Warrington, UK). The device is equipped with an ultrasensitive microbalance that measures weight change as the humidity is modified in the sample chamber at a constant temperature. After the samples were placed in a special container, they were dried at 25 °C in flowing nitrogen (250 mL/min) until their weights were in equilibrium at a relative humidity of less than 1%. Then, the relative humidity was gradually increased from 0 to 90%, in 10% humidity steps, with a pre-established equilibrium time between 40 and 60 min, the sorption equilibrium being obtained for each step. The relative humidity was then decreased, and desorption curves were registered.

#### 3.3.4. Mercury Intrusion Porosimetry

The mercury porosimetry analysis technique is based on the intrusion of mercury into a porous structure under stringently controlled pressures up to 33,000 psia or more. The AutoPore V 9605 Mercury Porosimeter (Micromeritics, Norcross, GA, USA) allows accurate determination of specific parameters for the characterization of the morphology of porous structures (total pore volume, total pore surface area, median pore diameter and sample densities).

#### 3.3.5. Swelling Degree

The swelling degree of the porous crosslinked and zwitterionic microparticles was determined in water (pH = 5.5) using the gravimetric method. Thus, 0.1 g of dried porous crosslinked or zwitterionic microparticles were immersed in 10 mL water, at 25 °C, for 24 h. The swelling degree was calculated using Equation (4):

(4) $$S_{W}\left(\%\right)=\frac{W_{s}-W_{d}}{W_{d}}\cdot 100,$$

where: W_S_—amount of swollen porous crosslinked or zwitterionic microparticles (g); W_d_—amount of dry porous crosslinked or zwitterionic microparticles (g).

### 3.4. Antibacterial Activity

The antibacterial activity of samples was determined against two reference strains, namely *Staphylococcus aureus* ATCC25923 (*S. aureus*) as Gram-positive bacteria, and *Escherichia coli* ATCC25922 (*E. coli*) as Gram-negative bacteria. All microorganisms were stored at −80 °C in 20% glycerol. The bacterial strains were refreshed on nutrient agar (NA) at 37 °C for 24 h. The antimicrobial susceptibility test using liquid broth medium was conducted via a slightly modified protocol of Kun and Marossy [48]. Microbial suspensions were prepared with the previously refreshed cultures in sterile solution to obtain turbidity optically comparable to that of 0.5 McFarland standards into 96-well tissue culture-treated plate. Plates with samples and inoculums (30 mg/mL concentration) were incubated for 24 h at 37 °C, under stirring at 250 rpm. Control microorganisms were incubated only with culture medium. The antimicrobial activity of samples after incubation with the microorganisms was assessed by MTS assay using the CellTiter 96^®^ AQueous One Solution Cell Proliferation Assay (Promega, Madison, WI, USA), according to the manufacturer instructions. After 23 h, the samples were removed from the plates and MTS reagent was added 1 h prior to absorbance readings. After the formazan formation, the final reading was performed at 490 nm on a FLUOstar^®^ Omega microplate reader (BMG LABTECH, Ortenberg, Germany). Experiments were conducted in triplicate and treated cell viability was expressed as percentage of control cells’ viability. Graphical data were expressed as means ± standard error of the mean.

### 3.5. Adsorption and Release Studies of TCH

Studies on adsorption of TCH onto G_40_N_30_T_30_ and G_40_N_30_T_30_-ZW microparticles were carried out in static conditions as follows: A quantity of 0.1 g of microparticles with known humidity was introduced into Erlenmeyer flask. Then, 10 mL of TCH solutions with concentrations between 0.25 × 10^−3^ and 1 × 10^−3^ g/mL (pH = 5.5) was added to the microparticles under gentle stirring at 180 rpm (Thermostated Shaker Bath M00/M01, Memmert, Schwabach, Germany) at 25 °C. After 8 h, the G_40_N_30_T_30_ and G_40_N_30_T_30_-ZW microparticles were removed quantitatively from the TCH solution by centrifugation (Janetzki T23 centrifuge, Heinz Janetzki KG, Leipzig, Germany) at 1000 rpm for 10 min. The concentration of TCH in the solution, before and after sorption, was determined using a UV-VIS Spectrophotometer (UV-VIS SPEKOL 1300, Analytik Jena, Jena, Germany) at the wavelength of 276 nm based on the calibration curve determined for the concentration range 0.5–0.36 × 10^−5^ g/mL.

The amounts of TCH at equilibrium, q_e_ (mg/g), and at any time, q_t_ (mg/g), were calculated from Equations (5) and (6), respectively:

(5) $$q_{e}=\frac{\left(C_{0}-C_{e}\right)\cdot V}{w},$$

(6) $$q_{t}=\frac{\left(C_{0}-C_{t}\right)\cdot V}{w},$$

where: C_0_—initial concentration of TCH solution (mg/g); C_e_ and C_t_—concentration of TCH at equilibrium and any time, respectively (mg/g); V—volume of TCH solution (L); w—weight of G_40_N_30_T_30_ and G_40_N_30_T_30_-ZW microparticles (g).

The release of TCH from porous crosslinked or zwitterionic microparticles was achieved by the systems containing the highest amount of loaded drug (initial TCH solution concentration of 1 × 10^−3^ g/mL). Thus: 100 mg of microparticles loaded with TCH were placed in 10 mL buffer solution of pH = 1.2 for 2 h and then in pH = 7.4, and stirred at low speed (50 rpm) in a thermostated bath (Thermostated Shaker Bath M00/M01, Memmert, Schwabach, Germany) at 37 °C. At certain time intervals, a known volume of the supernatant was extracted and the amount of released TCH was determined by UV-VIS spectrophotometry at 269 and 363 nm, respectively, using calibration curves calculated at pH = 1.2 and pH = 7.4. Subsequently, the same volume of fresh buffer solution (pH = 1.2 or pH = 7.4) was added to the release medium to complete the initial volume. The cumulative amount of TCH released (Q%) was calculated according to Equation (7) [49]:

(7) $$Q\left(\%\right)=\frac{10\cdot C_{n}+3\cdot \sum_{i=1}^{n-1}C_{i}}{M}\cdot 100,$$

where M—total mass of TCH adsorbed onto porous crosslinked and zwitterionic microparticles; C_n_ and C_i_—the concentration of TCH released from the microparticle–TCH systems in the buffer solutions determined at different times.

## 4. Conclusions

In this study, new porous microparticles with zwitterionic structure were synthesized by a two-step process as follows: (1) synthesis of the porous crosslinked microparticles based on GMA, NVI and TEGDMA by suspension polymerization reaction; (2) betainization reaction of porous crosslinked microparticles with sodium monochloroacetate. The chemical transformation brought a series of changes that includes increases in oxygen amount on the surface of the microparticles detected by EDAX analysis, increases in the values of specific surface area investigated with dynamic water vapor sorption and mercury porosimetry, increases of the value of pore volume and porosity, and decreases in pore size. Both G_40_N_30_T_30_ and G_40_N_30_T_30_-ZW microparticles were loaded with TCH, the latter being able to adsorb a higher amount of the antibiotic. The release capacity of TCH from microparticles was studied at pH = 1.2 (for 2 h) and then at pH = 7.4. The diffusion exponent, n, from Korsmeyer–Peppas equation, ranging 0.61–0.64 for both types of microparticles suggests that the TCH release mechanism is controlled by both diffusion and swelling processes. Preliminary studies suggest that zwitterionic porous microparticles have the ability to retain drugs and may act as candidates for sustained drug delivery systems. Moreover, the microparticles proved to have remarkable antibacterial activity against both Gram-positive and Gram-negative bacterial strains. However, in order to certify this statement, more in-depth studies must be carried out regarding pharmaceutical potential and biomaterial quality.

## Figures and Tables

**Table 1:** Porous structure parameters.

Sample Code	Specific Surface Area S_sp_ (m^2^/g)		Pore Volume (mL/g)	Porosity (%)	Mean Pore Diameter (nm)	Swelling Degree (%)
	Hg	DVS				
G_40_N_30_T_30_	48.32	289.13	1.314	46	109	261
G_40_N_30_T_30_-ZW	85.80	395.40	1.837	51	86	378

**Table 2:** Kinetic release parameters of drug release from G_40_N_30_T_30_ and G_40_N_30_T_30_-ZW microparticles loaded with TCH.

Sample Codes	Higuchi Model		Korsmeyer–Peppas Model			Baker–Lonsdale Model	
	k_H_ (min^−1/2^)	R^2^	k_r_ (min^−1/2^)	n	R^2^	k_BL_ × 10^3^ (min^−1/2^)	R^2^
G_40_N_30_T_30_-TCH	0.092	0.982	0.040	0.609	0.994	1.170	0.992
G_40_N_30_T_30_-ZW-TCH	0.118	0.986	0.026	0.644	0.995	0.805	0.996

k_H_, k_r_ and k_BL_—corresponding release rate constant for each described model; n—diffusion exponent.
